# Housing and Epigenetic Age Acceleration in the United States: Results From a Nationally Representative Sample

**DOI:** 10.1093/geroni/igaf122.4132

**Published:** 2025-12-31

**Authors:** Tongyu Gu, Jennifer Ailshire

**Affiliations:** University of Southern California, Los Angeles, California, United States; University of Southern California, Los Angeles, California, United States

## Abstract

Housing tenure is associated with a number of health outcomes at older ages, with homeowners typically demonstrating better health than renters. The underlying biological mechanisms linking housing to aging, especially in community-dwelling older adults, have yet to be explored. Using data on DNA methylation from the 2016 Health and Retirement Study Venous Blood Subsample, we examine the associations between housing tenure and epigenetic age acceleration in a nationally representative sample of US community-dwelling adults aged 56 + (n = 3,449). Survey-weighted linear regression models were used to predict epigenetic age acceleration measured with the following clocks: PhenoAge, GrimAge, PCGrimAge, and DunedinPACE. Compared to homeowners, renters showed age acceleration as measured by GrimAge (β = 0.56, p < 0.05), PCGrimAge (β = 0.49, p < 0.05), and DunedinPACE (β = 0.02, p < 0.05), though not by PhenoAge (β = 0.55, p = 0.26), after adjusting for sociodemographic factors (e.g., chronological age, gender, race/ethnicity, education, non-housing wealth, and number of household members) and health behaviors (e.g., smoking, alcohol use, obesity, and physical activity). Additional analyses did not find differences between homeowners with or without mortgages, or between homeowners and those who lived rent-free with others. These findings suggest renters experience earlier biological aging, which may increase aging-related morbidity and mortality risks. Homeownership is an important dimension of health equity among U.S. older adults.

